# A simple extension of the commonly used fitting equation for oscillatory structural forces in case of silica nanoparticle suspensions

**DOI:** 10.3762/bjnano.9.101

**Published:** 2018-04-05

**Authors:** Sebastian Schön, Regine von Klitzing

**Affiliations:** 1 Stranski-Laboratorium, Department of Chemistry, Technical University of Berlin, Strasse des 17. Juni 124, 10623 Berlin, Germany; 2 Soft Matter at Interfaces, Department of Physics, Technical University of Darmstadt, Alarich-Weiss-Strasse 10, 64287 Darmstadt, Germany

**Keywords:** confinement, depletion, fitting, silica nanoparticles, structural forces

## Abstract

**Background:** The ordering of molecules or particles in the vicinity of a confining surface leads to the formation of an interfacial region with layers of decreasing order normal to the confining surfaces. The overlap of two interfacial regions gives rise to the well-known phenomenon of oscillatory structural forces. These forces are commonly fitted with an exponentially decaying harmonic oscillation as introduced by Israelachvili (Israelachvili, J. N. *Intermolecular & surface forces;* Academic Press: San Diego, CA, USA, 1985). From the fit three important parameters are obtained, namely wavelength, amplitude and decay length, which are related to the period, the strength and the correlation length of the oscillatory structural forces, respectively. The paper addresses structural forces between a silica microsphere and a silicon wafer across silica nanoparticle suspensions measured with a colloidal probe AFM. Using the simple fitting procedure with three parameters often leads to underestimation of actually measured forces. The deviation of the fit from the experimental data is especially pronounced at small distances of the confining surfaces and at high concentrations of silica nanoparticles. As a consequence, the parameters of the common fit equation vary with the starting point of the fit. Although the wavelength is least affected and seems to be quite robust against the starting point of the fit, all three parameters show distinct oscillations, with a period similar to the wavelength of the oscillatory structural forces themselves. The oscillations of amplitude and decay length, which are of much higher magnitude, show a phase shift of 180° implying not only a dependence on the starting point of the fit but also on each other. The range affected by this systematic deviation of the fit parameters is much larger than the optically perceived mismatch between fit and experimental data, giving a false impression of robustness of the fit.

**Results:** By introducing an additional term of exponentially decaying nature the data can be fitted accurately down to very small separations and even for high silica nanoparticle concentrations (10 wt %). Furthermore wavelength, amplitude and decay length become independent of the starting point of the fit and in case of the latter two of each other. The larger forces at small separations indicate a more pronounced ordering behavior of the particles in the final two layers before the wall. This behavior is described by the proposed extension of the common fit equation.

**Conclusion:** Thus, the extension increases the accessible data range in terms of separation and concentration and strongly increases the accuracy for all fitting parameters in the system studied here.

## Introduction

Oscillatory structural forces are a genuine feature observed for simple and complex fluids in the vicinity of smooth surfaces [1–2]. Due to the ubiquitous nature of this phenomenon it has implications for a broad range of applications in microfluidic devices, for the stability of colloidal suspensions, or in a biological context, e.g., transport processes between biological surfaces or crystallization of proteins. Confined molecules or particles, aggregates or micelles tend to form a well-ordered layer in close proximity to a confining surface (henceforth called wall) with a density that is higher than that of the bulk. Since the molecules cannot penetrate each other, this layer of increased density is bordering on a region of lower density of molecules, compared to the bulk. The degree of ordering decreases exponentially with increasing distance from the wall, levelling off to bulk distribution. This region of oscillating density, also called the interfacial region, encompasses only a small number of layers. The thickness of the interfacial region, protruding into the surrounding fluid, is the multiple of the number and the distance between the layers. The distance between the layers, in case of uncharged system equals the molecular, particle, aggregate or micelle diameter [1,3]. If two interfacial regions are brought to overlap by approaching two surfaces the resulting density within the confinement will depend on the exact wall-to-wall distance. The oscillating density between the walls compared to the bulk leads to a changing, measurable pressure or force acting on the confining walls [1–2]. It can be measured using a variety of instruments, e.g., surface force apparatus (SFA) [2,4–6], thin film pressure balance (TFPB) [7–11], total internal reflection microscope (TIRM) [12–16], optical tweezers [17] or colloidal probe atomic force microscope (CP-AFM) [18–21]. Oscillatory forces have been observed in molecular liquids [1–2], but also in complex fluids such as liquid crystals [22–23], micellar or polyelectrolyte solutions [3,24–29] and particle suspensions [19,30–33]. Recent studies showed that for colloidal suspensions of charged silica nanoparticles the period of the oscillations is not related to the particle dimension as in case of molecular fluids or uncharged particles. Instead it matches well with the wavelength describing the asymptotic behavior of the bulk pair correlation function [34]. Moreover, it has been shown that this wavelength, which is interpreted as the distance between the particles within the confinement, is solely dependent on the particle number density and is equal to λ = *c^−^*^1/3^ [35]. This dependence seems fundamental as it is very robust against multiple parameters such as salt concentration, particle size [36], surface elasticity, roughness [37] and or potential [38], or addition of varying surfactants [39].

Oscillatory structural forces can be described either theoretically via the solutions of numerical simulations [34,40–42] or statistical mechanics equations [43–50] or with a semi empirical approach [51–53]. A common example for the latter is an exponentially decreasing harmonic oscillation:

[1]
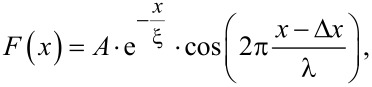


where *F* is the force, *x* is the separation between the walls, *A* is the amplitude of the oscillations and describes the particle interaction strength, ξ is the decay length and is related to the range of ordering of the particles normal to the wall, λ signifies the wavelength or period of the oscillation and characterizes the inter-particle distance, and Δ*x* refers to a phase shift. The theoretical descriptions and the semi-empirical approach have in common that they were designed for diluted samples and large separations. Despite this it has been proven that their validity extends towards medium separations [35,48,50,54]. Trying to fit data at small separations, such as the first and second layer, especially at higher concentrations of particles, remains a challenge. Including the first two layers into the fit leads to a poor fit of the experimental data at larger separations. It has been noted also, that amplitude *A* and decay length ξ vary strongly depending on the exact starting point of the fit. In this work, we focus on the dependence of the three fitting parameters *A*, ξ and λ with respect to the starting point of the fit region, as well as the introduction of a second exponential term into Equation 1. This additional exponential term in the description of the force curves accounts for the deviations between fit and data at small separations. The dependence of the fitting parameters onto the starting point with the extended equation is determined and compared with the previous results at varying nanoparticle concentrations. Recently, other groups also started using extended fit equations to describe oscillatory structural forces in different systems. The group of Borkovec, namely Moazzami-Gudarzi et al. [55–56], in case of strong polyelectrolytes between two micrometer-sized silica spheres and the group of Perkin, namely Alexander M. Smith et al. [57] and Samuel W. Coles et al. [58], for mixtures of an ionic liquid with a polar solvent using SFA. Both attributed the additional term to electrostatic double-layer forces. The systems investigated by both groups differ from the silica nanoparticle suspension studied in this work. In case of the Perkin group the oscillatory structural forces occur in a molecular system with hard sphere interactions. The group of Borkovec worked with charged objects (i.e., polyelectrolytes) but at high ionic strengths, which means strong electrostatic screening. The dissociation of the strong polyelectrolytes leads to a high ionic strength and thus a very small Debye length (*<*10 nm). This results in double-layer forces declining to zero even before the onset of the oscillatory structural forces. The pure silica nanoparticle suspension, as investigated in the present study, has a very low ionic strength, with Debye lengths of 25–50 nm. This would expand the range affected by potential double-layer forces well into the range where the oscillatory structural forces occur. Our work shows the impact of an extended fit equation in case of oscillatory structural forces between charged particles in case of low ionic strength, i.e., with an overlap of double-layer and oscillatory structural forces.

## Experimental

### Materials

Charged silica nanoparticles are used as the model system of an aqueous colloidal suspension. The stock solution was prepared via dialysis of LUDOX TMA-34 solution in a benzoylated dialysis tube (both Sigma Aldrich) for ten days with daily exchange of water (Milli-Q grade). The silica nanoparticles have a diameter of σ ≈ 26 ± 2 nm [34]. Silicon wafers (Wacker Chemie) were used as substrates.

### Preparation

The silicon wafers were cleaned prior to each experiment by etching in a 1:1:5 solution of hydrogen peroxide (30% Th. Geyer GmbH & Co KG), ammonium hydroxide (30–33% Carl Roth GmbH & Co KG) and water at 60 °C for 10 min (RCA method) [59]. The substrates were then rinsed extensively with water and dried in a nitrogen stream. Afterwards they were stored in a closed vessel in isopropanol (99.5% Geyer). Both, wafers and cantilevers were cleaned using an oxygen plasma (Diener electronics Femto) for 20 min immediately before use.

### Methods

Experiments have been conducted using the colloidal probe atomic force microscopy technique (CP-AFM) as introduced by Ducker and Butt [60–61]. For this method, a large silica sphere, 6.7 μm in diameter, is glued (UHU endfest 300) onto the tip of a cantilever (CSC38 tipless micromash) serving as colloidal probe. The spring constant of the cantilever was determined via the thermal noise method [62]. The surface of the colloidal probe and the silicon wafer form the two confining walls for the experiment. As the colloidal probe is orders of magnitude larger than their distance, the forces between them can be considered as forces between two parallel walls (Derjaguin approximation) [63].

Experiments were carried out with the MFP3D (Asylum Research) AFM using a starting distance of 1 μm, an approach and retraction velocity of 100 nm·s^−1^ and a sampling rate of 2 kHz. Preliminary results show an increased variation for the amplitude (24.4%) and the decay length (14.5%) for experiments conducted with different cantilevers/colloidal probes (nine measurements) compared to experiments conducted with the same cantilever/colloidal probe (five measurements), where the amplitude varied by 7.2% and the decay length by 2.6%. The differences are related to changes in the contact area of the colloidal probe and/or errors in the determination of the cantilever spring constant. Hence, the wavelength is unaffected by this. For this reason all experiments were conducted with a single cantilever. Another way to solve this problem is to measure the contact area of each cantilever used. Since the same colloidal probe was used for all experiments, all data are presented as force vs separation, as measured, without the need to normalize to the curvature radius of the colloidal probe. Force curves have been measured for suspensions with concentrations of nanoparticles ranging from 2 to 10 wt %. Wafer and cantilever were completely immersed in the sample suspension using a custom-built liquid cell.

**Figure 1:**
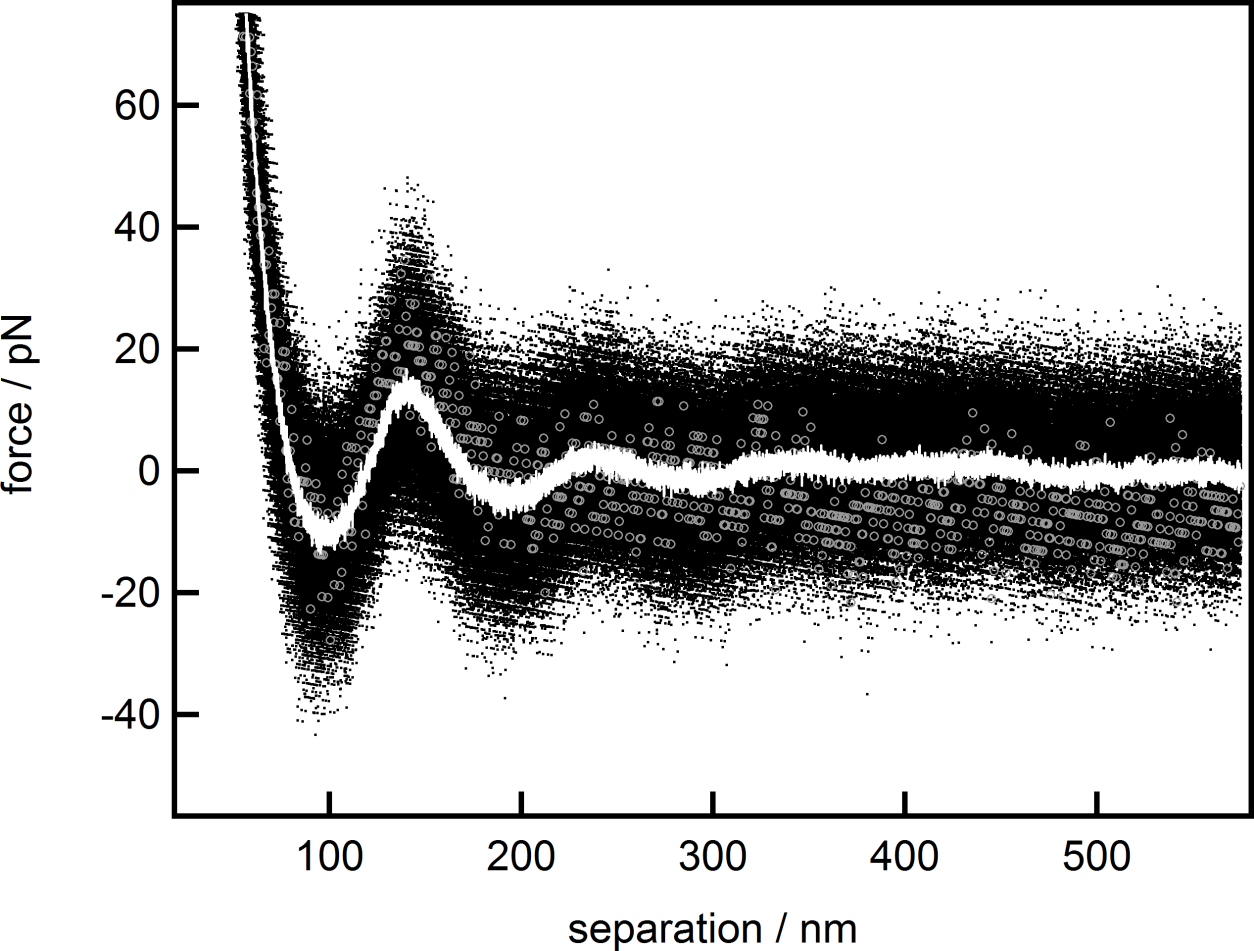
Force versus separation curve for the 2 wt % suspension. Grey open circles show a single force curve (number of data points reduced by a factor of 10 for better visibility), with only a single discernible maximum of the oscillatory structural force. Black dots show the combination of 67 individual measurements (1.3·10^6^ data points) with two detectable maxima. White dots represent the binominal smooth (width 10^3^ points) of the combined data showing a third maximum.

For each suspension 100 force curves were recorded as deflection (electric tension) in volts over the movement of the *z*-piezo in nanometers. The data were analyzed as follows: 1) Force and separation were calculated using the sensitivity slope of the linear contact region and the spring constant of the cantilever, which is determined from the thermal spectrum. 2) All force curves were aligned in relation to the *y*-axis using a linear fit of the force data at large separation, individually for each curve. 3) All force curves were aligned in relation to the *x*-axis using the contact region of the force curve that has already been used to determine the sensitivity. 4) All 100 force curves were combined into a preliminary set of combined force data. Combination here implies that each data point of each force curve is included into a single data set, resulting in a single force curve. No further individual alignment beyond the steps described above was performed. 5) Each single force curve was compared visually to the data set of combined force curves and in case of large deviations, in shape or phase shift, excluded. 6) The force curves that passed this check, 50 on average, showed a very homogenous appearance and have been combined into one final master curve. The combination of multiple force curves into one master curve greatly reduces noise and allows for the analysis of signals with very low strength as in case of suspensions with low concentrations shown in Figure 1.

The impact of this method is shown by comparing the significance of a single force curve with the combined master curve. The single force curve (open grey circles) only shows one maximum of the oscillatory force, ranging from 100 to 200 nm, while the rest of the data are too noisy to detect any meaningful signal. By combining 67 individual force curves into one master curve (black dots, 1.3·10^6^ data points) a second maximum can be discerned in the range from 200 to 300 nm. A third one, extending from 300 to 400 nm, is made visible by applying a binominal smoothing filter with a width of 10^3^ data points (white line). The noise reduction due to the combination of data leads to an increase in the accessible signal range from 100 to 400 nm instead of only up to 200 nm as in case of the single force curve. This range contains three full periods of structural oscillations compared to only one for the single force curve. This enables an efficient fitting of the data which otherwise would not have been possible.

**Figure 2:**
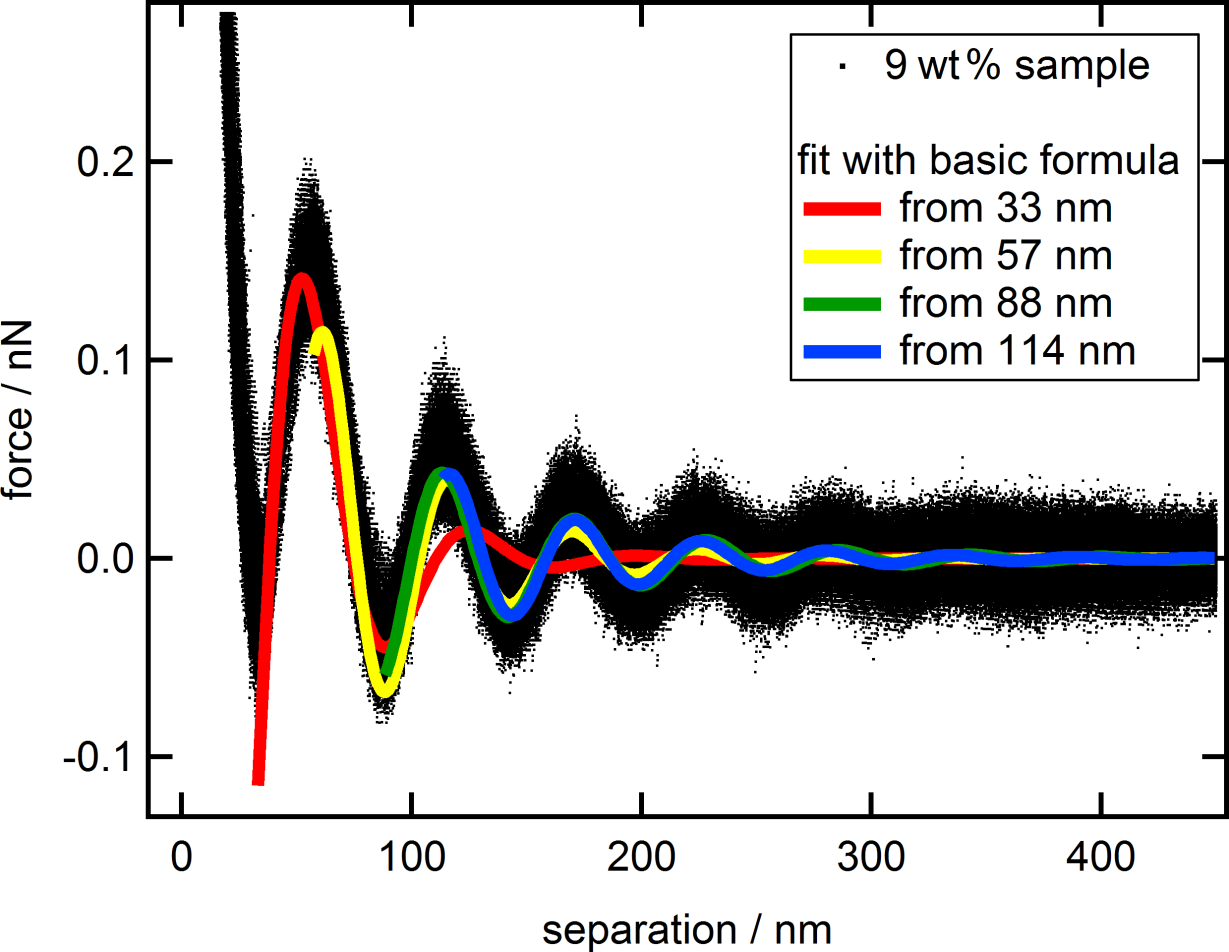
Combined force data of the 9 wt % suspension (black dots) together with fits based on Equation 1 from different starting points: 33 nm (red), 57 nm (yellow), 88 nm (green) and 114 nm (blue).

It is known that Equation 1 is, strictly speaking, valid only for large separations [47]. Nevertheless, it has already been shown that the experimental data are well described down to medium separations, apart from highly concentrated suspensions, where a deviation between fit and measured data can be observed [34]. This observation is confirmed in Figure 2, where fits from different starting points of the force over separation data for the 9 wt % suspension are displayed.

**Figure 3:**
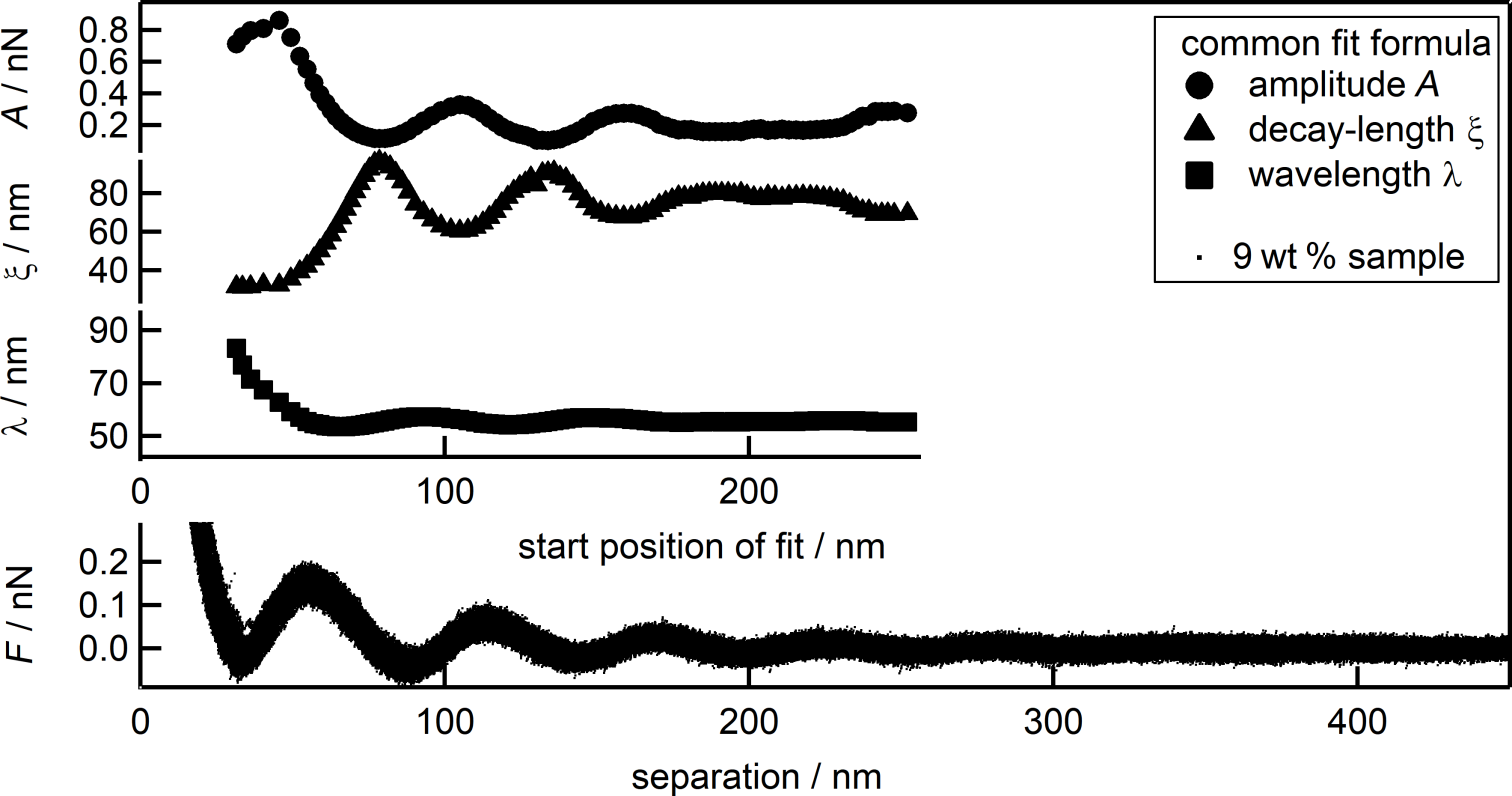
Combined force data of the 9 wt % suspension (black dots) together with the fit parameters *A* (circles), ξ (triangles) and λ (squares) as a function of the separation or the starting point of the individual fit, respectively.

The fit starting already at the first minimum (red) deviates strongly at large separations, while all other fits represent the data reasonably well. Fits starting at or after the second minimum (green and blue) seem almost congruent and show only minor deviations towards each other. For a better understanding about where the fits start to deviate from the data, a detailed analysis of the fitting behavior with respect to the starting point has been conducted. For that purpose, the data have been fitted multiple times with the fit region extending from the starting point, at the first minimum, gradually changing towards larger separations to the end at 600 nm. The individual resulting fit parameters: amplitude *A*, decay-length ξ and wavelength λ are shown as functions of the corresponding starting point of the fit (Figure 3).

**Figure 4:**
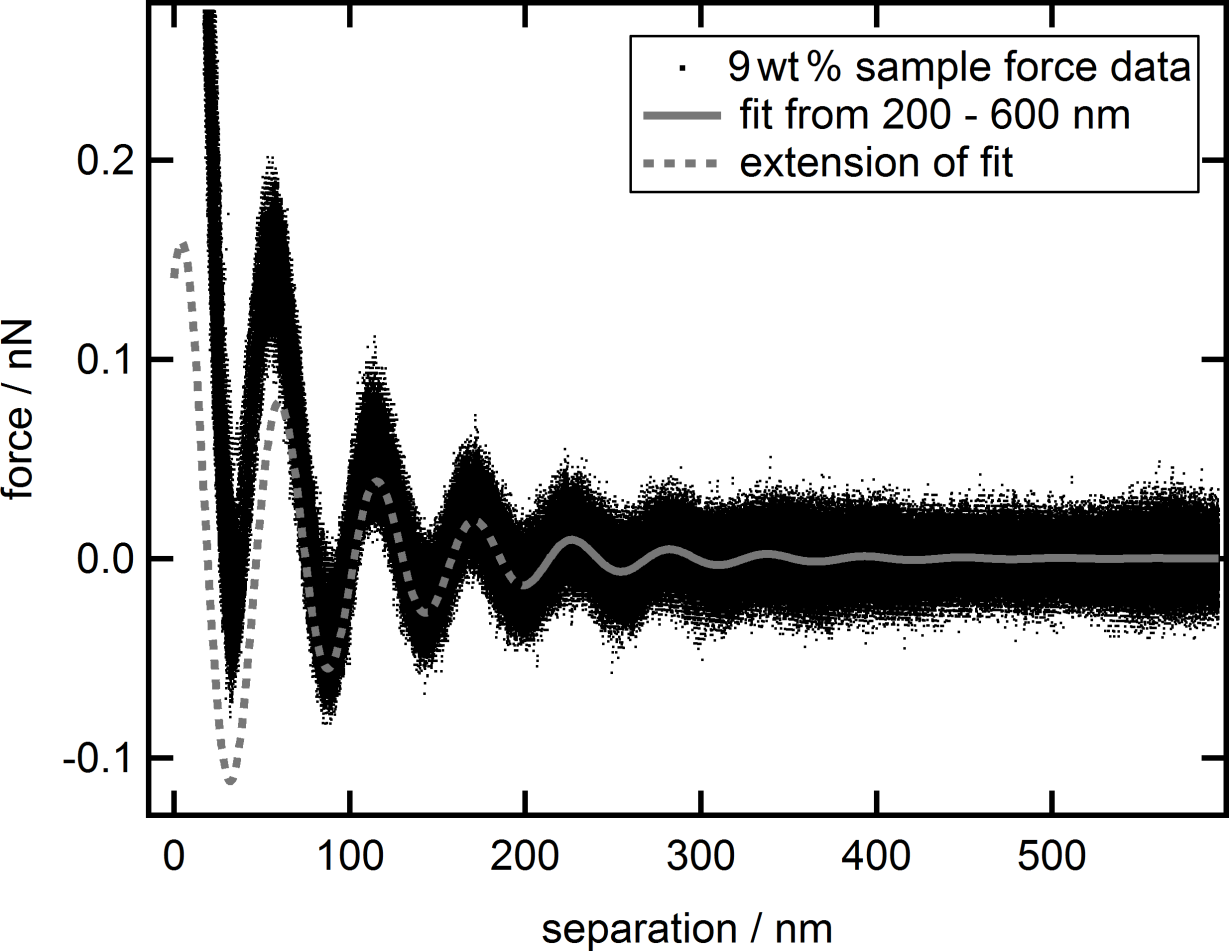
Combined force data of the 9 wt % suspension (black dots) together with fit based on Equation 1 made from 200 to 600 nm (solid grey line) and extension of that fit towards smaller separations (dashed gray line).

The largest deviations for all three parameters occur within the first period of oscillation starting at the first minimum of the force at 33 nm up to approximately 60 nm, which is shortly after the first maximum. Figure 2 showed that the fits after the second minimum, at approximately 90 nm can be hardly distinguished from each other. Despite this, pronounced oscillations of both, amplitude and decay length with a phase shift of 180° can be observed in the range of 60 to 180 nm, which is well beyond the third minimum. Additionally, it becomes apparent that both parameters are dependent on each other, as the oscillation of one parameter with the variation of the fit starting point is mirrored by the other with a phase shift of 180°. The wavelength is much more robust, showing only a slight variation for starting points of the fit larger than 60 nm. The oscillations of the wavelength are of much lower magnitude and with a different phase shift compared to the amplitude and decay length, emphasizing the independence of λ from *A* and ξ. The period of those deviations, 53 nm for the amplitude, 55 nm for the decay length and 56 nm for the wavelength, are very similar and close to the average of the wavelength 56 ± 4 nm, which is actually the period of the oscillatory structural forces itself. For separations of 180 to 230 nm all three parameters seem to remain constant. Figure 4 displays the fit made at a starting point of 200 nm (solid gray line) and the extrapolation towards smaller separation (dashed gray line).

Although the fit precludes the first three full periods, the extrapolation towards zero separation represents the data reasonably. Only at the first period from 30 to 90 nm the force values resulting from extrapolation are below the experimental data. The same effect was noticed for various measurements of silica nanoparticle suspensions at different concentrations, with higher concentrations tending to show larger deviations. This generated the idea to extend the common fit equation by an additional term of repulsive nature that only affects small separations. Inspired by the additional terms shown by Israelachvili [51] for hydration forces near to a hydrophilic surface, a simple decaying exponential function was chosen leading to the new extended Equation 2:

[2]
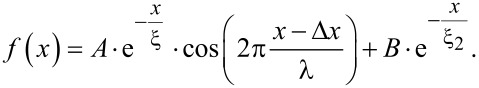


**Figure 5:**
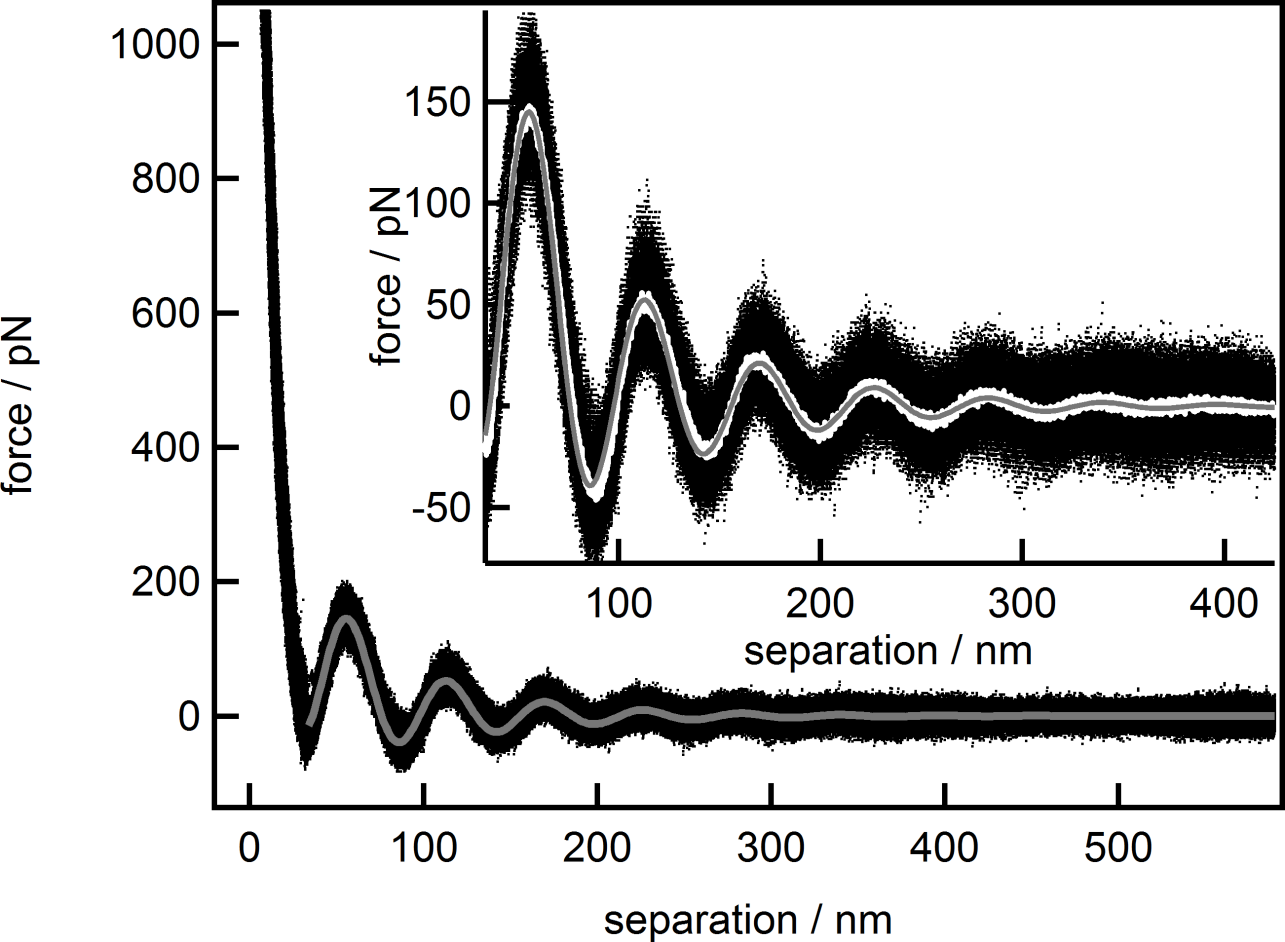
Combined force data of the 9 wt % suspension (black dots) together with the fit based on Equation 2 made from 34–600 nm (solid gray line). The inset displays the binominal smooth (solid white line, 10^3^ data points) of the data.

A fit of the data with this extended equation is shown in Figure 5.

**Figure 6:**
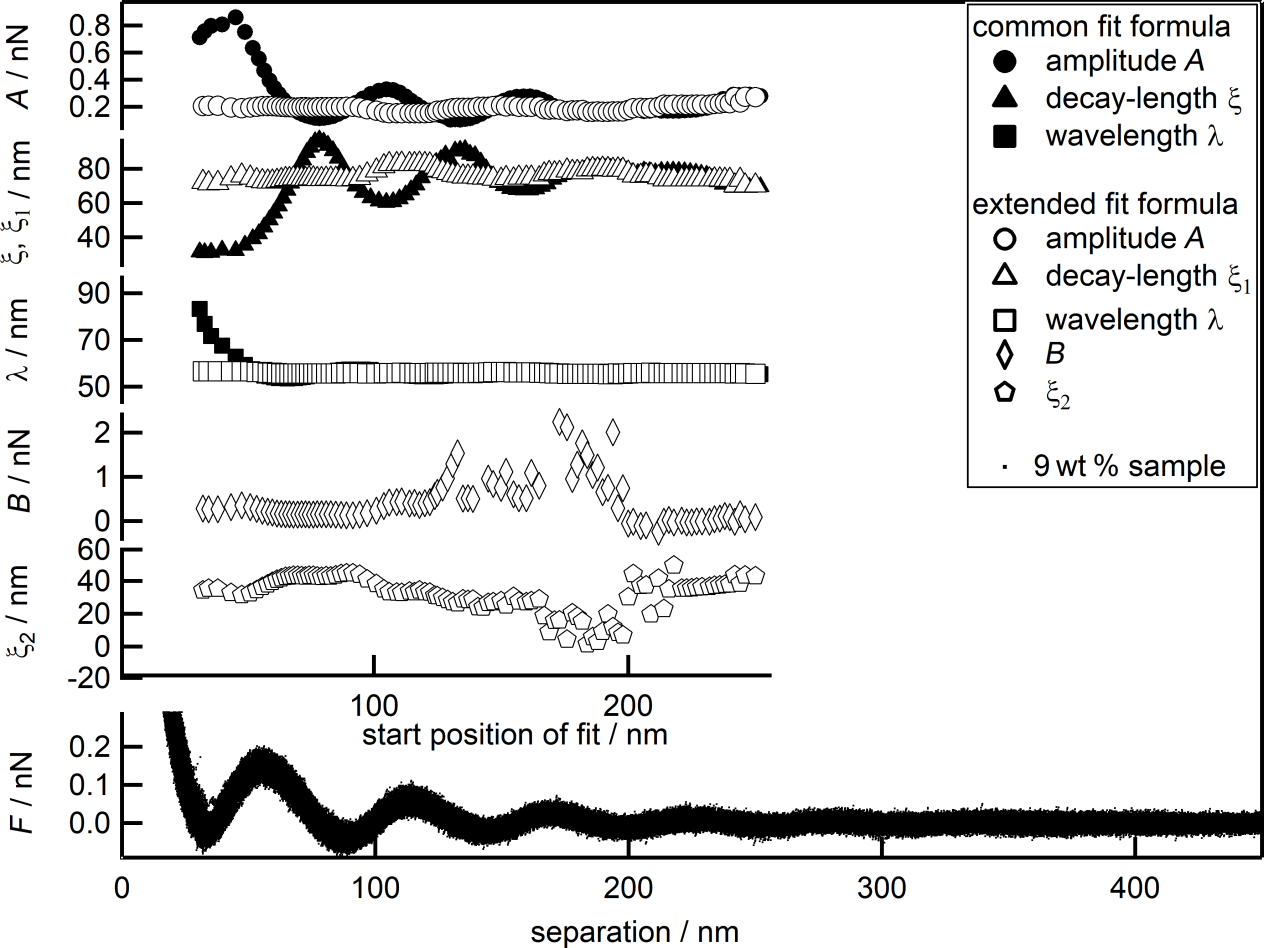
Combined force data of the 9 wt % suspension (black dots) together with the fit parameters over the separation or the starting point of the individual fit, respectively: *A* (circles), λ (squares) and ξ, ξ_1_ (triangles), *B* (diamonds) and ξ_2_ (pentagons). Open symbols represent parameters fitted with the new equation, while full symbols represent the data from Figure 3 for ease of comparison.

The fit (solid gray line), starting at the first minimum, matches the force data (black dots) very well at small as well as at large separations. The high quality of the fit becomes evident when comparing with the smoothed data (solid white line) as both almost coincide. Of course, an increase in fitting quality is to be expected when two new parameters have been introduced in the fitting function. Separation-dependent analysis of the force data, like the one shown in Figure 3, has been carried out utilizing the extended Equation 2. In the first analysis, all parameter from Equation 2 were set as free parameters, and the results are given in Figure 6. As the differences between the decay lengths, as derived with the common and the extended equation, are of special concern and will be discussed below, they are named individually. The decay length derived from the common equation will henceforth be named as ξ, while the decay length from the extended equation will be named as ξ_1_ for the first term and ξ_2_ for the newly introduced second term.

Figure 6 shows force data for the suspension with 9 wt % (black dots), the three standard parameters *A* (circles), ξ, ξ_1_ (triangles), λ (squares) and additionally the two new parameters *B* (diamonds) and ξ_2_ (pentagons) in dependence of the separation or the starting point of the fit, respectively. Foremost, the oscillatory behavior of all three standard parameters is subdued. Furthermore, the large deviations of all three parameters, in the range from the first minimum of the force up to a separation of 60 nm, are suppressed. Moreover, the large variance of all three parameters over the whole range of starting points of the fit is strongly reduced. This makes them independent of the starting point of the fit and much more reliable. The values obtained for *A*, ξ and λ at large separations, with the common equation, are very similar compared to the ones found with the extended equation over the whole range of fitting. This behavior is to be expected and confirms that the deviation between common fit and experimental data is limited to small separations. Furthermore, it shows that the two new parameters are well suited and sufficient to describe the deviation. The two new parameters *B* and ξ_2_ themselves show relative small variance at small separations up to 120 nm, while after that point the data scatter over a broad range with the blanks representing data points outside the scale. The scale has not been increased to include those data points to achieve a reasonable resolution for the parameters at small separations. The large scattering especially beyond 120 nm can be explained by the fact, that the additional parameters have been introduced to describe the short-ranged deviation as observed in Figure 4, especially at separations below 100 nm. It is therefore to be expected that they are not suited for fitting at larger separations.

In a second analysis (Figure S1, Supporting Information File 1), the number of free fit parameters is reduced by setting *B* and ξ_2_ as constants. For the constant value the average of *B* and ξ_2_, in the range from 30–120 nm from the first analysis, is taken. The results of the second analysis for the three main parameters *A*, ξ_1_ and λ resemble strongly the results of the first analysis. As *B* and ξ_2_ are constants in the second analysis and both show variation in the first analysis, it is prudent to determine whether the average values of *B* and ξ_2_ are the best choice to describe the data.

**Figure 7:**
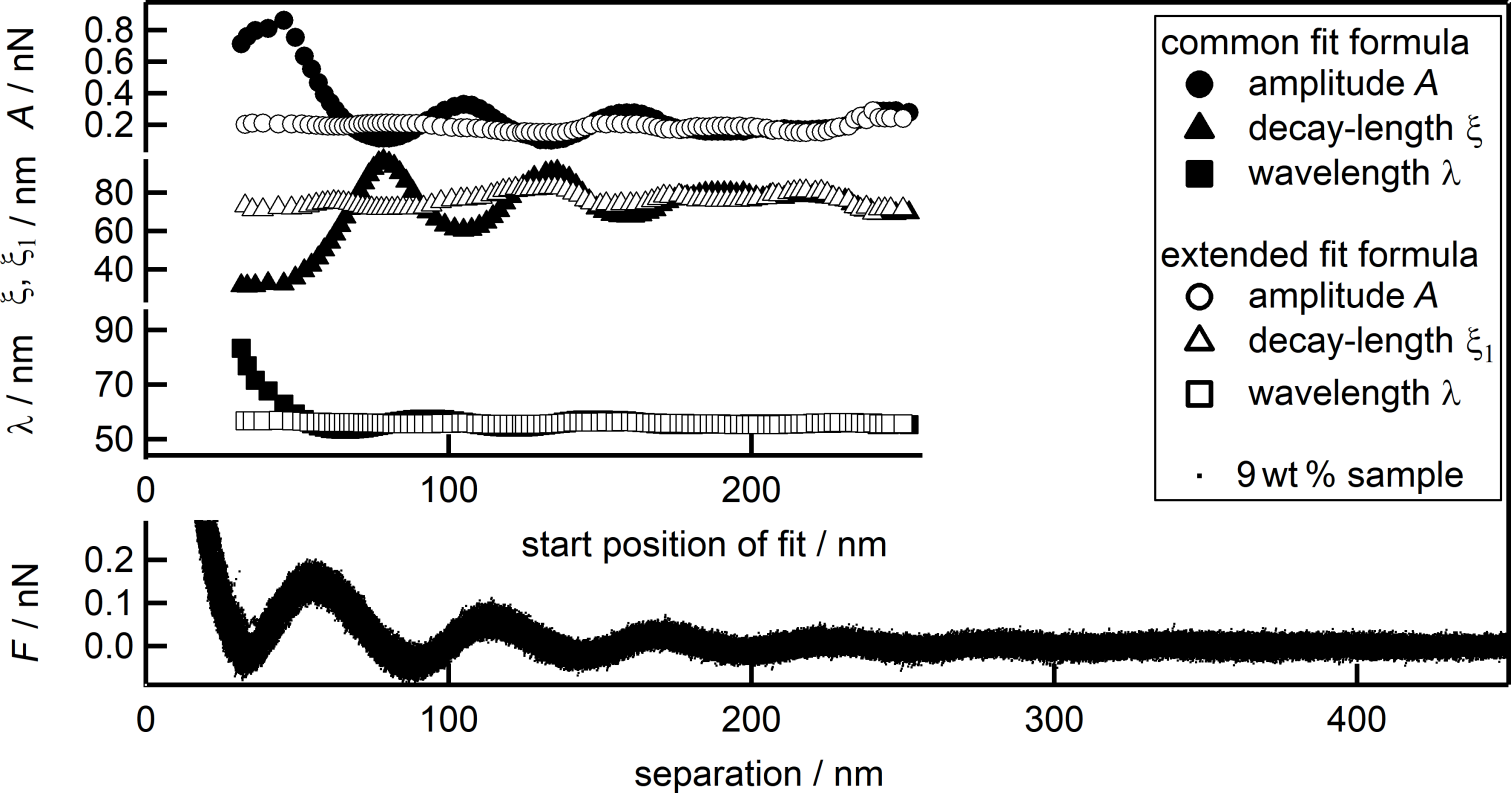
Combined force data of the 9 wt % suspension (black dots) together with the fit parameters over the separation or the starting point of the individual fit, respectively: *A* (circles), ξ, ξ_1_ (triangles) and λ (squares). Open symbols represent parameters fitted with the new equation and the best combination of *B* and ξ_2_ being kept constant, while full symbols represent the data from Figure 3 for ease of comparison.

In a third analysis, the best combination of *B* and ξ_2_ are to be found. This can be achieved by using an iterative approach, where *B* and ξ_2_ are varied, forming a 2D matrix with decreasing mesh size with increasing number of iterations (Figure S2, Supporting Information File 1). As criterion for the best combination of *B* and ξ_2_, the minimum of the combined relative errors of *A*, ξ_1_ and λ was utilized (Figure S3, Supporting Information File 1). For more details see Supporting Information File 1. The result of the third analysis is shown in Figure 7.

Figure 7 shows the dependence of *A* (circles), ξ, ξ_1_ (triangles) and λ (squares) on the starting point of the fit together with the force data of the 9 wt % suspension as a function of the separation. As values of the new parameters *B* and ξ_2_ the best set determined by iteration has been used. They have been kept constant and are not displayed. The emerging picture concerning the three standard fit parameters strongly resembles the one found during the first analysis shown in Figure 6. The large deviations of *A*, ξ_1_ and λ on the starting point of the fit, compared to the fit with the common equation, in the range from 33 to 60 nm, as well as the oscillations in the range of 60–180 nm have vanished. The pronounced oscillations, with a phase shift of 180°, of amplitude and decay length as observed when using the common equation are strongly reduced in case of *A* and ξ_1_ obtained with the extended equation. The data scatter much less showing very similar values over the whole range of the fit for all three parameters. The fit parameters are independent of the starting point of the fit and of each other. Finally, these values equal the values obtained for *A*, ξ and λ obtained with the common equation at large separations. This demonstrates clearly that *B* and ξ_2_ can be kept constant to gain the positive effects on *A*, ξ_1_ and λ, as observed in Figure 6. This underlines the validity of the newly introduced extension to the common fit function. The results as shown so far, for the 9 wt% suspension, are in principal observed for a broad range of concentrations. The oscillatory behavior of the three standard parameters, when using the common Equation 1, is usually more pronounced for higher concentrated suspensions. In the following, a comparison of the common and the extended fit equation is given in Table 1 for all concentrations measured. Therefore, the relative errors 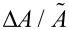, 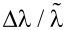 and 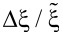 were calculated for both methods by dividing one standard deviation (Δ) by the corresponding average (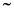).

**Table 1:** Comparison of the relative errors of *A*, ξ and λ obtained by using the common or the extended fitting equation. In the last column, the relative increase in precision of the extended equation over the common equation is given by dividing the error of the common equation by the error of the extended equation. The quotient (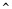) is given for all three parameters.

wt %	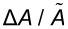		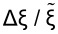	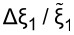	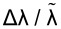		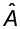	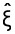	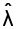
	common	extended	common	extended	common	extended			

2	0.566	0.313	0.134	0.098	0.020	0.015	1.8	1.4	1.3
3	0.386	0.302	0.117	0.098	0.026	0.017	1.3	1.2	1.5
4	0.713	0.215	0.209	0.074	0.036	0.013	3.3	2.8	2.8
5	0.812	0.261	0.218	0.089	0.038	0.009	3.1	2.5	4.3
6	0.840	0.348	0.250	0.089	0.052	0.009	2.4	2.8	6.0
7	1.079	0.226	0.246	0.085	0.054	0.014	4.8	2.9	3.8
8	0.399	0.157	0.135	0.062	0.024	0.006	2.5	2.2	3.7
9	0.670	0.138	0.205	0.046	0.074	0.006	4.9	4.5	12.3
10	1.319	0.134	0.254	0.059	0.099	0.006	9.8	4.3	16.1

In the last columns, the quotients of the relative errors of the common equation divided by the extended equation, for amplitude (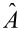), decay length (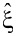) and wavelength (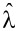) are displayed. These quotients show the increase in precision when using the extended equation for the fitting. The trend for the gain in precision is not linear but seems to be strongest for λ, followed by *A* and ξ_1_, with the highest concentrated nanoparticle suspension showing the largest increase in precision. Generally, for suspensions with low concentrations, the precision of the fit increases by a factor of 1.5 to 3.0, with some exceptions. Highly concentrated suspensions show increased precision by a factor of 5 to 10 or above. Furthermore, the relative errors 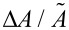, 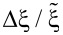 and 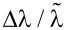 increase with concentration when using the common fit equation. However, these errors all show a decreasing trend when applying the new extended Equation 2. This behavior seems natural as the oscillatory structural forces are more pronounced for higher concentrated suspensions. Thus, the data contains more information. The increase in information includes the deviation between common fit and data resulting in an increase in uncertainty of the standard fit parameters. Contrary to that, the extended fit equation is well suited to describe the whole profile of the oscillatory structural forces. Therefore the increase in information here leads to an increase in precision.

## Results and Discussion

**Figure 8:**
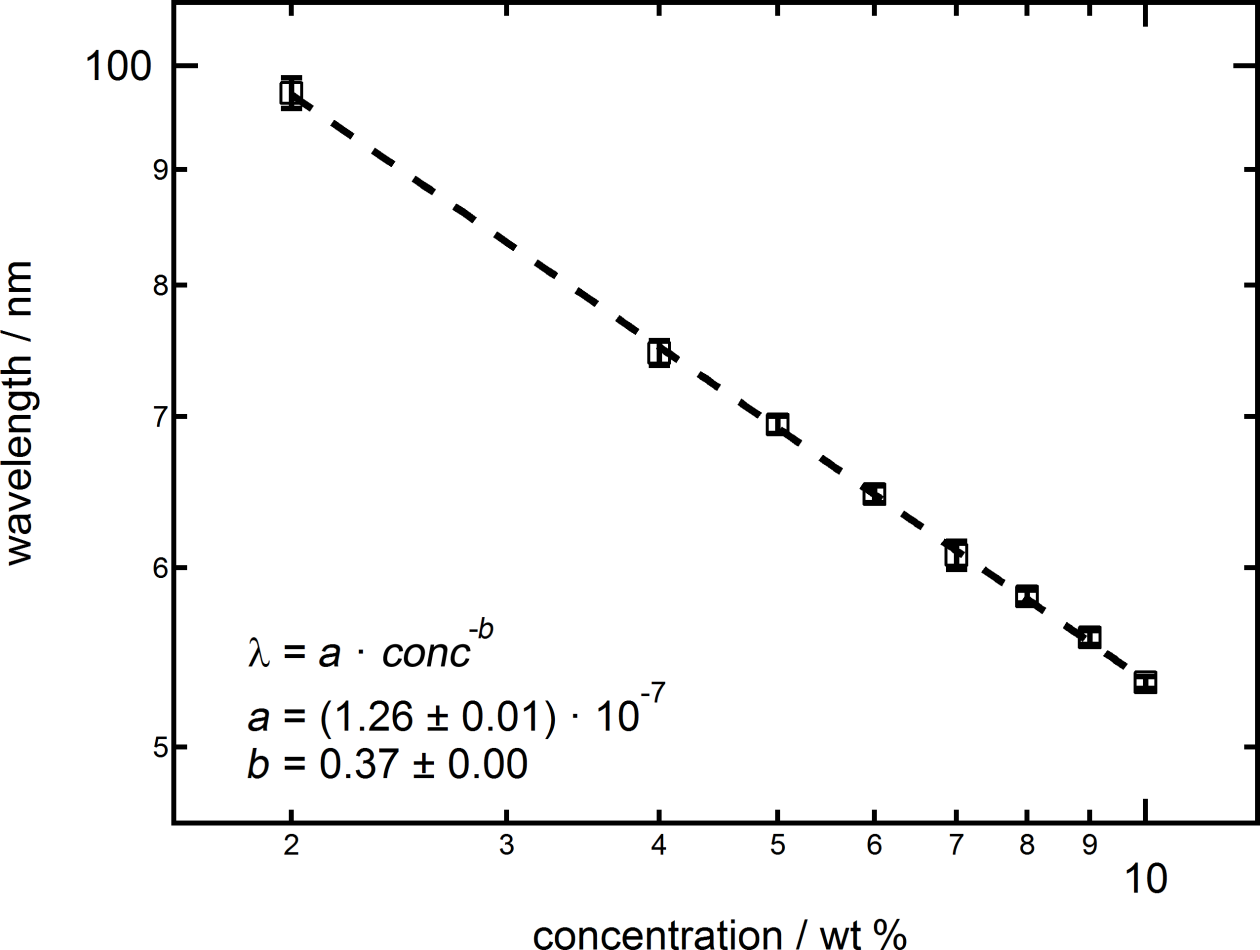
Log–log scale of wavelength λ as a function of the silica nanoparticle concentration and the respective fit by a power law.

Figure 8 shows the wavelength λ, representing the distance between the layers of particles in the confinement, plotted as a function of the concentration of the suspension. The dependence of the wavelength of the concentration of the silica nanoparticle suspension follows a clear power law. The exponent of *b* = 0.37 as extracted from the fit λ = *a*·*conc^−b^* is very close to the ideal value for the average particle distance in the bulk *b* = 1/3. The prefactor *a* relates the concentration in wt % to the number particle density. This confirms the findings of Klapp et al. [34] and Zeng et al. [35] that the average distance between the particles in confinement is solely dependent on the particle concentration of the suspension. Several publications in the past claimed that for charged particles the period scales with 2(*R* + κ^−1^) including the particle radius *R*. The Debye length κ^−1^ is calculated from the ion concentration induced by the silica nanoparticle number density assuming a charge per particle of *Z* = 35 [7,9,27]. Zeng et al. already mentioned that this is not the general case and only valid for certain particle concentrations and ionic strengths.

**Figure 9:**
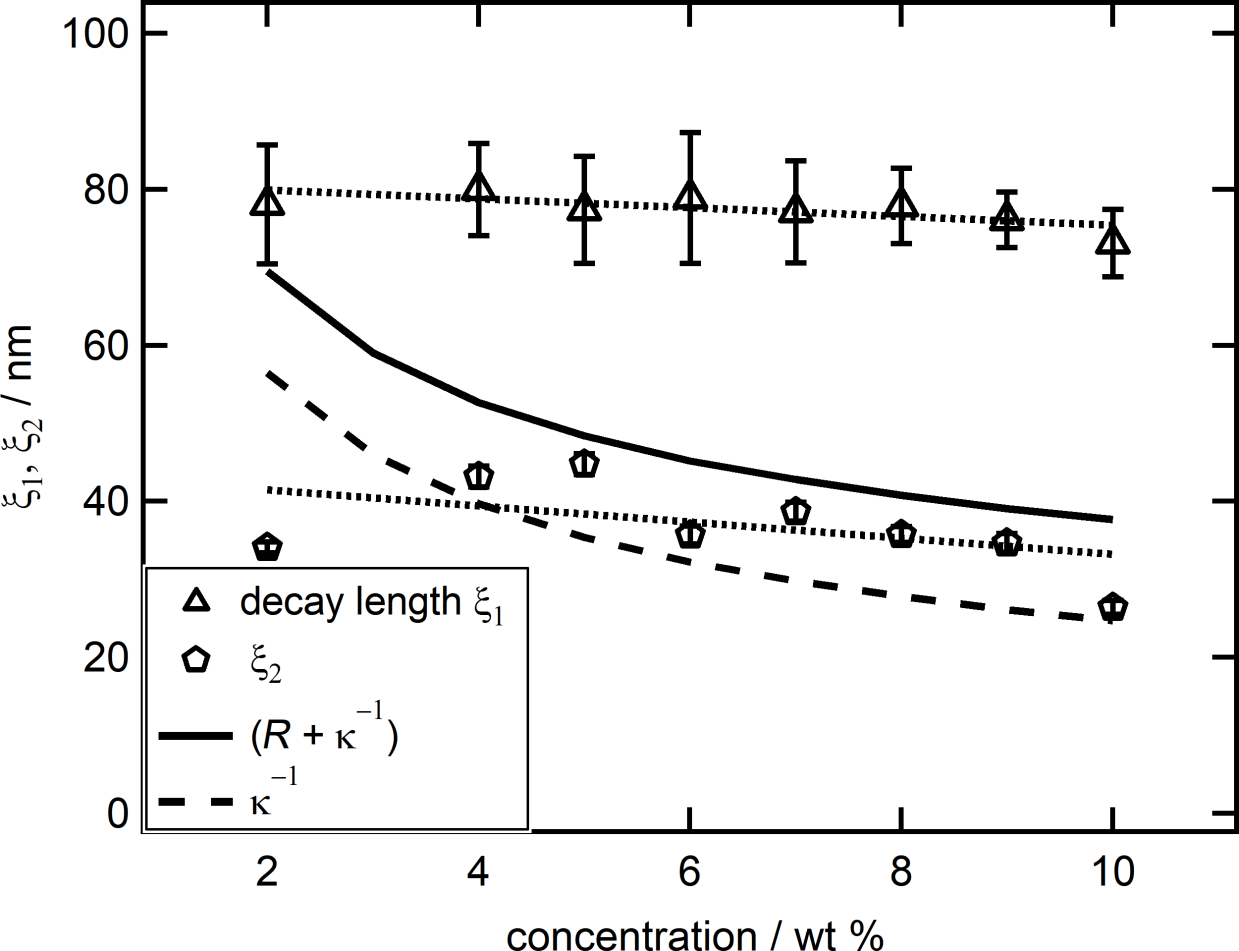
Decay length ξ_1_ (triangles) together with ξ_2_ (pentagons) in nm as a function of the suspension concentration in wt % together with linear trends (dotted lines). Also shown are the Debye length κ^−1^ (dashed line) and the sum of Debye length and particle radius κ^−1^ + *R* (solid line).

Figure 9 presents the decay length ξ_1_ (triangles), as well as ξ_2_ (pentagons), in dependence of the suspension concentration. The data for the decay length ξ_1_ is best represented by a linear fit (dotted line). The slightly negative slope indicates only a light dependence on the nanoparticle suspension concentration and/or a materials parameter related to it. However, due to the small value of the slope in relation to the error bars a constant behavior of ξ_1_ cannot be excluded. Concerning ξ_2_ a similar progression is observed. The emerging linear trend is still decreasing, but with a slightly larger slope, again a constant behavior of ξ_2_ independent of the suspension concentration cannot be excluded. However, both slopes are much smaller than one would expect from the increase in ionic strength caused by increasing the concentration of silica nanoparticles (κ^−1^ curve).

The decrease of ξ_2_ with increasing particle concentration would support the interpretation by the groups of Borkovec and Perkin [55–58]. Their results indicate that the extension term of the fit describes the electrostatic repulsion between the confining surfaces and links ξ_2_ to the electrostatic screening length. A comparison with the calculated Debye length, according to the supporting information given by Zeng and co-workers [36], shows relative good agreement except for the lowest silica nanoparticle concentration of 2 wt %. Previous results supported a relation between the decay length ξ (as obtained with the common fit of Equation 1), the particle radius and the Debye length according to ξ = *R* + κ^−1^ [36]. To compare our results to these findings the sum of the calculated Debye length and particle radius (13 nm) is shown as black solid line in Figure 9. None of both decay lengths, ξ_1_ and ξ_2_, presented here coincide with ξ = *R* + κ^−1^. Instead the sum of Debye length and particle radius gradually decreases from values close to ξ_1_ at low silica nanoparticle suspension concentrations down to values approaching ξ_2_ at high concentrations. The deviation between the previous and the current findings is interpreted as follows: At low suspension concentrations, the deviation between common fit and measured data is small. Therefore, the part of the extended fit equation (Equation 2) designed to describe this deviation becomes small as well. This implies that for low suspension concentrations, as the deviation approaches zero, the extended fit equation converses into the common fit equation with ξ_1_ approaching ξ. For low concentrations, this is affirmed in Figure 9, as the value of the decay length ξ_1_, obtained with the extended fit equation, approaches the sum of radius and Debye length (*R* + κ^−1^), which in turn equals ξ, as found by Zeng et al. At large suspension concentrations the importance of the additional term in the extended fit equation becomes more important, as the deviation between common fit and measured data gets larger. Fitting the data with the common equation does not take this into account and leads to a different, much smaller ξ as can be seen in Figure 9. Under these circumstances the use of the common fit equation is an attempt to fit data that contains two decay lengths with an equation that only accounts for one. Ultimately this leads to a conversion of the decay length ξ, from values equal to ξ_1_ at small concentrations towards values approaching ξ_2_ at large concentrations.

**Figure 10:**
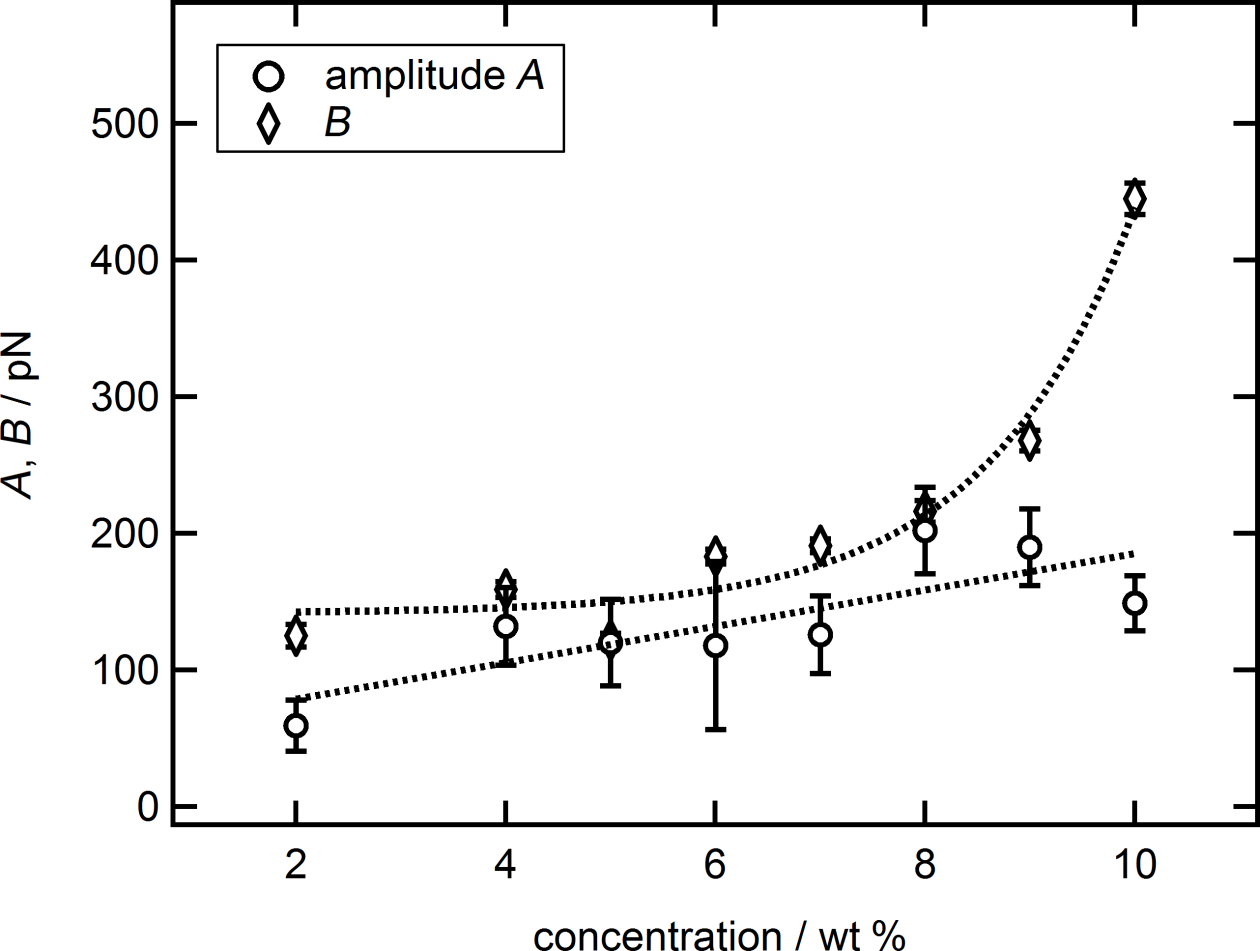
Amplitude *A* (circles) as well as parameter *B* (diamonds) in pN as a function of the suspension concentration in wt % together with trends (dotted lines). The large increase in *B* at higher concentrations matches with the observed deviation between experimental data and the common fit equation.

As shown in Table 1 the deviation between experimental data and common fit increases with increasing silica nanoparticle concentration, meaning that the integral of the extension term *B*·ξ_2_ increases as discussed below. Hence, the decrease of ξ_2_ has to be overcompensated by an increase of *B* with increasing suspension concentration as shown in Figure 10.

In contrast to this, the amplitude *A* of the common fit shows a trend of a linear increase with increasing silica nanoparticle concentration. The trend is not unambiguous as the data scatter. This is related to the high sensitivity of the amplitude to multiple factors. On one hand, identifying the factual *A* depends on the correct determination of the spring constant. As mentioned above this was done via the thermal noise method and already introduces an error of about 10%. On the other hand, it is highly sensitive to the correct transformation of the measured deflection over *z*-piezo movement, as received from the AFM, into the force as a function of the separation. Errors made in the transformation lead to shifts in the zero position on the separation axis of the force graph, thus affecting the exponential prefactors *A* and *B*. Although the combination of the data as reported above strongly reduces this error, it still gives an estimated inaccuracy of 5%. Also, the contact area of the colloidal probe is a major factor that influences *A* and *B*. Despite our efforts to remove this source of uncertainty by using only one colloidal probe through all experiments it cannot be ignored. Inverse scanning images of the colloidal probe before and after the measurements showed changes in the contact area due to silica nanoparticles sticking to the surface of the colloidal probe. Despite these problems, the linear increasing trend matches expectations of stronger pronounced oscillatory structural forces for larger concentrations. It also reaffirms the findings of Zeng et al. [36] in which a linearly increasing amplitude for silica nanoparticles, of different size, with concentration was also found.

The data for *B*, describing the strength of the deviation at small separations between the common equation and real data, is best described by an exponential trend. This matches with expectations and strongly underlines the importance of the newly introduced term in Equation 2, especially for highly concentrated suspensions. The strong increase in *B* conflicts with the interpretation that the newly introduced term represents double layer forces. In that case *B* would be related to surface properties of the confining walls and decrease with increasing ionic strength/particle concentration. It is difficult to attribute a correct error towards the parameters *B* and ξ_2_. Both parameters vary during the first separation-dependent fit analysis, indicating a given amount of uncertainty. During the final separation-dependent fit analysis both parameters were constants with no errors assigned to. The errors given in Figure 9 and Figure 10 have been calculated from the width of the last iteration step in the third analysis of the data, which was used to determine the best set of *B* and ξ_2_ (see Supporting Information File 1).

**Figure 11:**
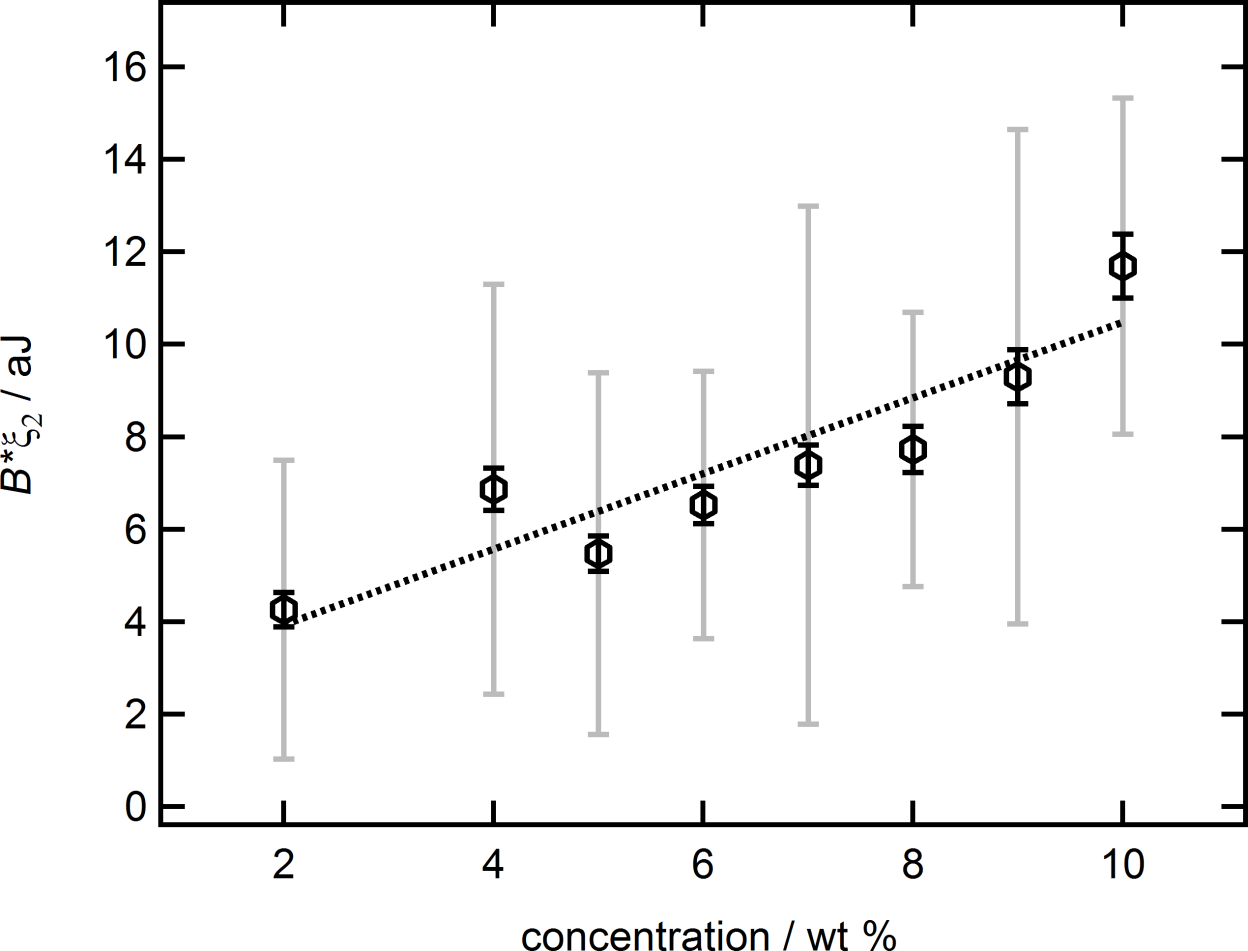
*B*·ξ_2_ in aJ as a function of the suspension concentration in wt % together with a linear trend. Two different errors are given, one based on the width of last iteration step (solid black bars) and the other on one standard deviation of free parameters from the first analysis with extended fit equation (grey solid bars), see Figure 6.

Taking a more detailed look at the decreasing ξ_2_ in combination with the exponentially increasing *B*, Figure 11 shows the product *B*·ξ_2_ (hexagons). This product, being the integral of the difference between common and new fit, describes the deviation energy *E*_deviation_ as measured at low separations compared to the common equation.

The errors of the deviation energy are calculated by adding the relative errors of *B* and ξ_2_. Resulting in a lower and an upper margin of error, depending on the source for the relative errors of *B* and ξ_2_. If the errors are taken from the width of the last iteration step, they are small (black solid bars). The problem here is that the errors depend solely on the number of iteration steps and can thus be artificially decreased by increasing the number of runs. Therefore, this represents a lower boundary for the uncertainty of the deviation energy. If the errors are taken from the standard deviation of the free parameters *B* and ξ_2_ during the first analysis with the extended fit equation they are much larger (solid grey bars). The problem with this approach is that it neglects the refinement of *B* and ξ_2_ achieved during the third data analysis with the iteration procedure. It therefore serves as upper boundary for the uncertainty of the deviation energy. The real error should be something in between. Although the data scatter, the linear trend (dotted line) is significant enough to demonstrate the increasing nature of the deviation energy with increasing silica nanoparticle suspension concentration. Furthermore, it shows that the general trend of the deviation energy, which lead us to the introduction of the additional term in Equation 2, is dominated by the parameter *B*.

As mentioned already, the superposition of oscillatory structural forces and an additional repulsive term has been investigated before. The group of Perkin investigated ionic liquids in polar solvents [57–58]. In the latter using the same type of generic formula for a damped oscillation with an additional exponentially decaying contribution as utilized by us (Equation 2). They linked the additional repulsive term with the double-layer forces and attributed the second decay length directly to the electrostatic screening length. The group of Borkovec [55–56] also used double-layer forces to describe deviations between experimental force data and oscillatory structural forces. The system consisted of two silica particles in an aqueous solution of a like-charged strong polyelectrolyte (NaPSS). The increase of the deviation energy with increasing silica nanoparticle concentration as it is found in the present study contradicts those findings. The ionic strength and thus the electrostatic screening in the nanoparticle suspension increases with concentration of nanoparticles. With increasing electrostatic screening, the contribution of the double-layer forces should decrease. The system as studied by the group of Perkin investigates oscillatory structural forces on a molecular scale. The interactions are hard sphere-like and no interaction with the double-layer forces is to be expected. The system as studied by the group of Borkovec is similar to ours, as both depend on electrostatic repulsion between charged entities. But with the high ionic strength in case of the polyelectrolyte solution the double-layer forces decline to zero even before the onset of the oscillatory structural forces. Thus, again no mutual effect between double-layer forces and oscillatory structural forces can be observed. It has been noted that in their paper [55] the superposition of double-layer force and oscillatory structural force fits the data very well before the first minimum of the respective force curve. From the first minimum onwards, a deviation between theoretical and experimental data remains especially at the position of the first minimum. This reinforces our findings for *E*_deviation_, as the extension to the common fit equation for the structural oscillation forces was designed to accommodate deviations between fit and experimental data over a large range of separation values beyond the first minimum of the force curve. The small electrostatic screening in the pure silica nanoparticle suspension used in this work extends the double-layer forces well into the range of the observed oscillatory structural forces. The observed increase in deviation energy could originate in an enhanced ordering of silica nanoparticles affected by the electrostatic repulsion from the confining walls. The range of this enhanced ordering would be determined by the electrostatic screening length, while the magnitude would be dependent on the concentration of particles in compliance to our experimental results.

## Conclusion

The introduction of an additional term of decaying exponential behavior to the common fit equation allows to extend the range of the fit towards very small separations. Even starting at the first minimum of the force curve, and including the whole first period, the fit represented the experimental data well over the whole range of separation values. Furthermore, a detailed analysis of the behavior of the three important parameters amplitude *A*, decay length ξ and wavelength λ in dependence of the starting point of the fit region showed, that using the common fit equation, all of them exhibit oscillations with a period resembling the wavelength. It shows, that all three parameters depend on the starting point of the fit. Although the wavelength remains independent of amplitude and decay length, the latter two are no longer independent fit parameters, as the behavior of one is mirrored by the other with a phase shift of 180°. The oscillations extend to large separations of up to 180 nm in case of a 9 wt % silica nanoparticle suspension, which is well into the third period of oscillation of the force data. Even more importantly, the oscillations of the parameters arise also in a range where the fits with the common equation can hardly be distinguished from each other, therefore giving a false impression of robustness against the starting point of the fit.

The application of the extended fit equation removes the large deviation of all parameters within the first period of the force data that arise when using the common equation. Moreover, it removes the oscillatory nature in dependence of the starting point of the fit for all three parameters. The fit becomes independent of its starting point. The increase in precision, i.e., the quotient of the relative accuracies of *A*, ξ and λ determined with the common and the extended equation, has been calculated. The precision increases by a factor of 1.5 to 3.0 for suspensions of low and medium concentration and 5 to 10 for suspensions above 8 wt %, with *A* and λ being more affected than ξ. Although the increase in precision is highest for λ, it must be noted that this parameter showed small absolute variance with the common equation already. The amplitude *A* profits the most from the use of the extended equation, with the relative error in case of the 10 wt % suspension decreasing from 130% with the common equation to 13%. The average values of all three parameters found with the extended equation resemble those obtained with the common equation at large separations. To summarize: All fit parameters become independent of each other and of the starting point of the fit as well as more precise.

It has been shown that the period of the force oscillations λ, representing the average particle distance perpendicular to the wall, strongly depends on the particle concentration, while the decay length ξ_1_ shows only a slight decrease with increasing concentration. The amplitude increases with increasing concentration. The deviation energy, describing the deviation between common and extended fit, is the product of the newly introduced parameters *B* and ξ_2_. The deviation energy increases with particle concentration, whereby the exponential increase of *B* with concentration dominates the negative correlation found for ξ_2_. While ξ_2_ exhibits similar values to the electrostatic screening length, the increasing *B* prohibits an identification of the additional repulsive term in Equation 2 with double-layer forces alone, as done elsewhere [55–58]. The interaction between double-layer forces and oscillatory structural forces could lead to an enhanced ordering of particles. Only particles within the range of the double layer forces would be affected, explaining the dependence of ξ_2_. While the strength of the enhanced ordering (*B*) would rely on the particle concentration.

## Supporting Information

File 1 Additional experimental data.
The Supporting Information explains in detail the iterative process used to find the best set of *B* and ξ_2_ to describe the force data with the extended equation.
