# Supplementary material for: A simple extension of the commonly used fitting equation for oscillatory structural forces in case of silica nanoparticle suspensions
Source: Beilstein J Nanotechnol. 2018 Apr 5;9:1095–107. doi: 10.3762/bjnano.9.101 (PMC5905248; doi:10.3762/bjnano.9.101)
Supplement: File 1 — Additional experimental data. The Supporting Information explains in detail the iterative process used to find the best set of B and ξ2 to describe the force data with the extended equation. [file Beilstein_J_Nanotechnol-09-1095-s001.pdf]

Supporting Information

for

A simple extension of the commonly used fitting equation for oscillating structural forces in case of silica nanoparticle suspensions

Sebastian Schön[1] and Regine von Klitzing*[2]

Address: [1]Stranski-Laboratorium, Department of Chemistry, Technical University of Berlin, Strasse des 17. Juni 124, 10623 Berlin, Germany and [2]Soft Matter at Interfaces, Department of Physics, Technical University of Darmstadt, Alarich-Weiss-Strasse 10, 64287 Darmstadt, Germany

Email: Regine von Klitzing - klitzing@smi.tu-darmstadt.de

* Corresponding author

Additional experimental data

In the present paper, we used a separation-dependent fit analysis to investigate the change of fit parameters with the common or extended fit equation in dependence of the starting point of the fit. The force data were analyzed with the common equation and oscillatory changes in A, ξ and λ were observed. After that, the same analysis was performed using the extended equation. In the first analysis with the extended equation, the new parameters B and ξ_2 were set as free parameters. As a consequence, the main parameters A, ξ_1 and λ scattered less and became independent of the starting point of the fit.

Second analysis method

In a second analysis, the number of free parameters during the fit was reduced, by setting B and ξ_2 constant. The results are shown in Figure S1.

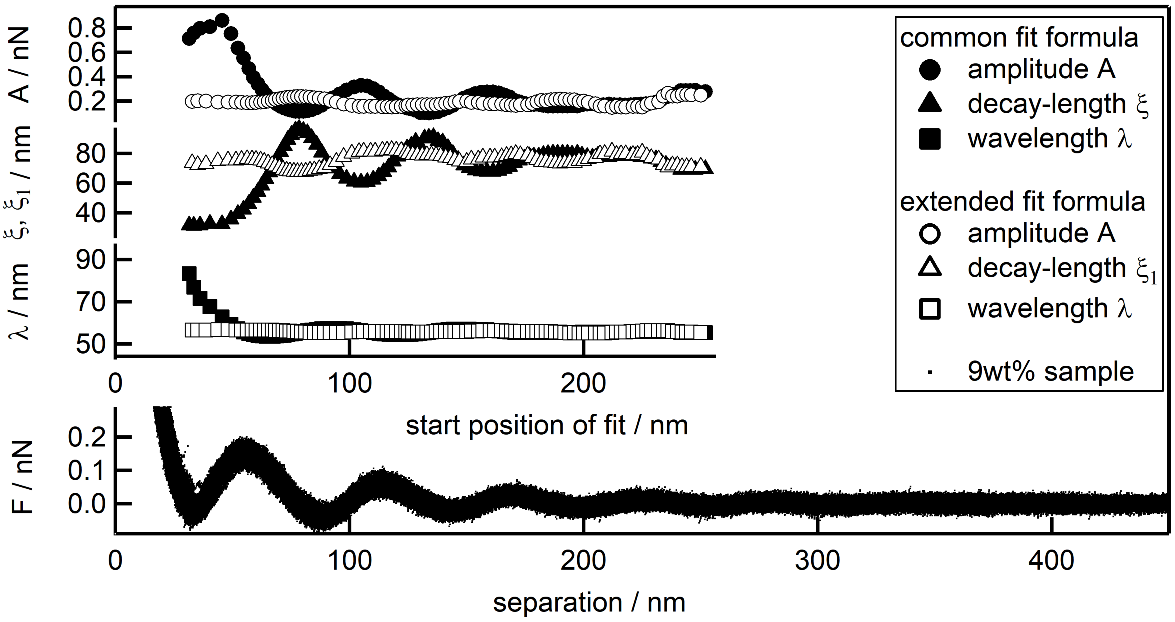

Figure S1: Combined force data of the 9 wt% suspension (black dots) together with the fit parameters over the separation respectively the starting point of the individual fit: A (circles), ξ,ξ_1 (triangles) and λ (squares). Open symbols represent parameters fitted with the new equation and fixed B and ξ_2. The values were taken from the average in the region with low variance during the first analysis (see Figure 6 of the main manuscript). Full symbols represent the data from the analysis with the common equation (see Figure 3 of the main manuscript) for ease of comparison.

B and ξ_2 were calculated by taking the average values from the first analysis in the separation region with low variance. In case of the 9 wt % silica nanoparticle suspension this included the values from 30 to 120 nm separation (see Figure 6 of the main manuscript). The results for the three standard parameters A (circles), ξ_1 (triangles) and λ (squares) strongly resemble the results from the first analysis (Figure 6 of the main manuscript). The oscillations in dependence on the starting point of the fit are suppressed and the large deviations for starting points of the fit at smaller separations than 60 nm are removed. Therefore, reducing the number of free parameters during the fit seems appropriate to avoid overfitting, while retaining the beneficial effect on the three standard parameters. Some uncertainty remains though, whether the resulting set of A, ξ_1 and λ is the best to describe the data, due to the scattering of B and ξ_2.

Third analysis method

A third analysis has been performed to reduce this uncertainty, using an iterative process to find the best set of B and ξ_2. The iterative process has been designed as follows: Figure S2.

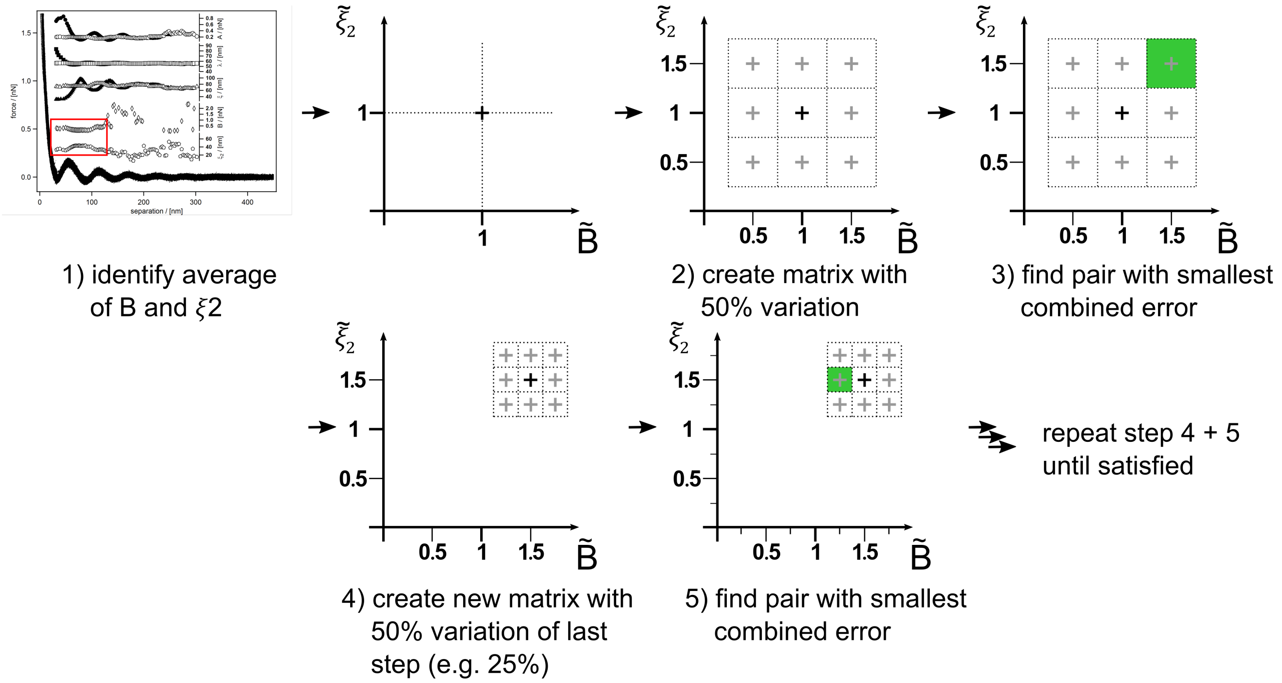

Figure S2: Description of the iterative process used to find the best set of B and ξ_2, which gives the smallest combined error for the main parameters A, ξ_1 and λ

The iterative process was initialized with the same set of B and ξ_2 as used in the second analysis, were both parameters were enforced as constants. In the next step, B and ξ_2 were varied by 50% to create a matrix of three values for B and ξ_2 each, for a total of nine different combinations. For each combination of B and ξ_2 a separation-dependent fit analysis was performed. To identify the combination of B and ξ_2 that is best suited to describe our data, we determined the minimum of the product of the relative errors of A, ξ_1 and λ. Usually relative errors are summed up to get a combined error. Multiplication was chosen in order to give each of the three parameters equal weight. Otherwise the relative error of A, which is the largest (see Table 1 in the main manuscript), would have dominated. The found pair of B and ξ_2 then served as new starting point for the next step in the iterative process. The variance for B and ξ_2 in the new step was set to 50% of the previous step, e.g., 25% in the second step, 12.5% in the third and so on. The iteration was stopped after five steps, which reduced the variation of B and ξ_2 in the final step to 3% of the initial value. The whole process was done automatically with a self-written program using the IGOR PRO software. The result of the iterative process in case of the 9 wt % silica nanoparticle suspension is shown in Figure S3.

As shown in Figure S3 the initial value of B and ξ_2 (black) is not far off from the final value after five steps of iteration (magenta). This shows that taking the average of B and ξ_2 from the first analysis, were both parameters have been free, has been a good choice. In fact, the first two steps of the iteration (blue and green) show no improvement at all. Only in the third step (yellow) a lower minimum for the combined error has been found, noticeable due to the shift, best seen in the top view, of the matrix of combinations of B and ξ_2 in the fourth step (orange). The results of the separation dependent fit analysis with the best set of B and ξ_2 (magenta) are shown in Figure 7 of the main manuscript. They strongly resemble the results of the second analysis as presented in Figure S1, but express lower variance of the three standard parameters A, ξ_1 and λ.

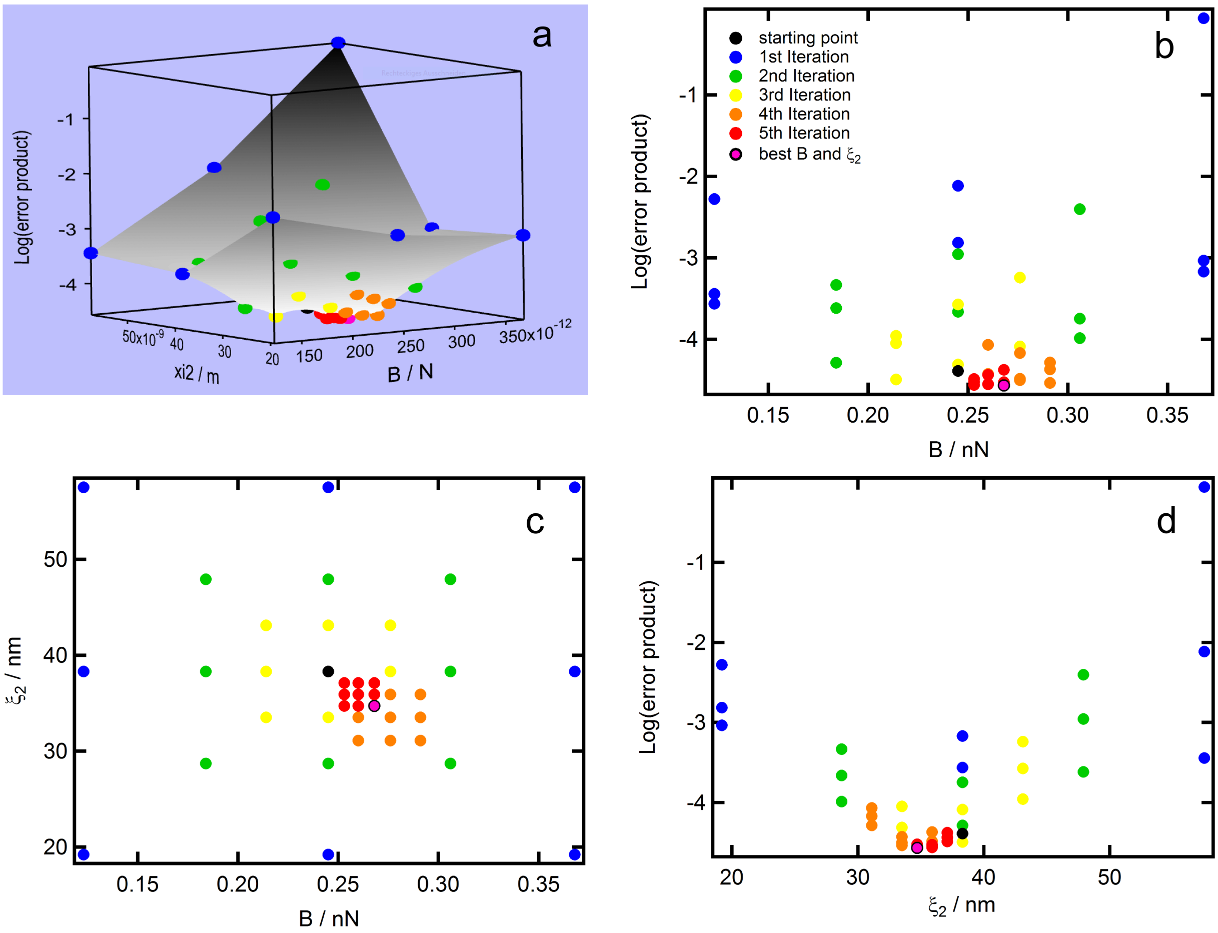

Figure S3: Results of the iterative process with the logarithmic value of the combined relative errors from A, ξ_1 and λ, over B and ξ_2 as 3D surface plot (a) and the corresponding side views Log(error) over B (b), Log(error) over ξ_2 (d) and the top view ξ_2 over B (c).
